# Precise deposition of platinum sub-nanoclusters on Cu-doped CeO_2_ for low temperature CO oxidation

**DOI:** 10.1038/s42004-020-00398-7

**Published:** 2020-10-30

**Authors:** Teresa S. Ortner

**Affiliations:** Communications Chemistry, https://www.nature.com/commschem

## Abstract

Platinum catalysts are widely employed for vehicle exhaust treatment, but their activity is poor when the catalyst is cold. Now, precise control of the interfacial structure of platinum sub-nanoclusters on copper-doped ceria delivers excellent activity at low temperatures.

The efficiencies of vehicle exhaust treatments are continuously being improved, but cold-start emissions remain a challenge. Now, a team led by Bin Shan and Rong Chen from Huazhong University of Science and Technology in Wuhan, China, achieve effective low-temperature catalytic CO oxidation through intricately anchoring Pt catalysts on fine-tuned Cu–O–Ce interfacial structures. With a customized atomic layer deposition method, they produce Pt sub-nanoclusters on optimized Cu-modified CeO_2_ supports, reaching an onset of CO oxidation reactivity at below room temperature and a turnover frequency of 0.26 s^−1^ at 80 °C (10.1038/s41467-020-18076-6)^1^.

Despite considerable progress made in developing low-temperature exhaust catalysts, the harsh working environment deteriorates high-performing catalysts, such as nano-Au or nanoparticulate Co_3_O_4_^2,3^. Hence, Pt-based catalysts are still the most widely used exhaust cleaning catalysts owing to their high reactivity and chemical stability. Strong metal–support interactions make reducible oxide-supported Pt catalysts, such as Pt_1_/PN-CeO_2_ or Pt-CeO_2_-Al_2_O_3_, prime candidates for good activities paired with sufficient stability. However, for 50% CO conversion, Pt_1_/PN-CeO_2_ requires at least 68 °C^4^ and Pt-CeO_2_-Al_2_O_3_, like most Pt-based catalysts, needs 150+ °C^5^. Chen and colleagues now achieve 50% CO conversion at a significantly lower temperature of 34 °C.

While atomic layer deposition (ALD) is widely used for catalyst preparation, the deposition of Pt atoms on oxide supports is sensitive to oxide surface structures and ALD protocols. Thus, it is a major challenge to simultaneously control the interfacial reactivity and the catalyst structure—especially in the size range of sub-nanometer clusters. The team has now evolved the ALD method into a redox-coupled process for intricate size control of the Pt catalysts (Fig. 1): an organo-Pt(IV) precursor is reduced in-situ and deposited besides metallic Pt on the support rods. “Our method yields Pt clusters with a narrow size distribution anchored on the surface of CeO_2_ and Cu-doped CeO_2_ supports, which results in different coordinate structures compared to atomically dispersed Pt catalysts”, explains Chen.

**Fig. 1:**
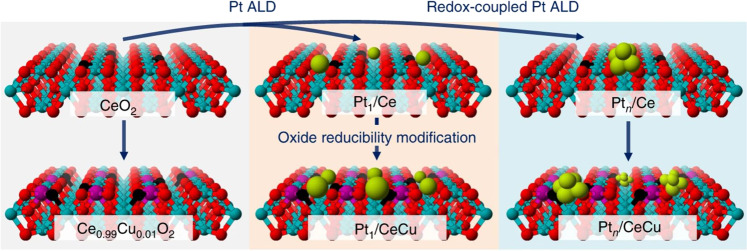
Schematic illustration of the preparation processes for supported Pt catalysts. The redox-coupled Pt atomic layer deposition (ALD) allows for intricate control of size as well as oxidation state of the catalyst. Ce, O, Pt, and Cu atoms shown as red, cyan, green, and purple, respectively. Reproduced from Springer Nature: *Nat. Commun*. **11**, 4240, copyright 2020.

The researchers additionally investigated the effect of low concentration Cu doping of the oxide support on low-temperature CO oxidation activity. “We found that Cu dopants, in such low concentrations as Ce_0.99_Cu_0.01_O_2_, can not only activate the interfacial oxygen, but also weaken the CO adsorption on supported Pt clusters”, Chen explains. “On the contrary, higher concentrations such as Ce_0.95_Cu_0.05_O_2_ can lead to the formation of CuO_x_ species on the surface of the CeO_2_ supports, which have negative effects on the activity of supported Pt catalysts”.

“We believe that our preparation strategy is generally expandable to the design of highly efficient noble metal based catalysts, which can be applied in various catalytic reactions, such as preferential CO oxidation in H_2_, water-gas shift reaction, elimination of volatile organic compounds, and electrocatalytic reactions as well”, concludes Chen.
